# Forensic tracers of exposure to produced water in freshwater mussels: a preliminary assessment of Ba, Sr, and cyclic hydrocarbons

**DOI:** 10.1038/s41598-020-72014-6

**Published:** 2020-09-22

**Authors:** Paulina K. Piotrowski, Travis L. Tasker, Thomas J. Geeza, Bonnie McDevitt, David P. Gillikin, Nathaniel R. Warner, Frank L. Dorman

**Affiliations:** 1 Department of Chemistry, The Pennsylvania State University, University Park, PA USA; 2 Department of Civil and Environmental Engineering, The Pennsylvania State University, 212 Sackett Building, University Park, PA 16802 USA; 3 Environmental Engineering, Saint Francis University, Loretto, PA USA; 4 EES-14, Los Alamos National Laboratory, Los Alamos, NM 87544 USA; 5 Department of Geology, Union College, 807 Union St, Schenectady, NY 12308 USA; 6 Department of Biochemistry, Microbiology and Molecular Biology, The Pennsylvania State University, 107 Althouse Lab, University Park, PA 16802 USA

**Keywords:** Biogeochemistry, Environmental sciences

## Abstract

Hydraulic fracturing is often criticized due in part to the potential degradation of ground and surface water quality by high-salinity produced water generated during well stimulation and production. This preliminary study evaluated the response of the freshwater mussel, *Elliptio complanata,* after exposure to produced water*.* A limited number of adult mussels were grown over an 8-week period in tanks dosed with produced water collected from a hydraulically fractured well. The fatty tissue and carbonate shells were assessed for accumulation of both inorganic and organic pollutants. Ba, Sr, and cyclic hydrocarbons indicated the potential to accumulate in the soft tissue of freshwater mussels following exposure to diluted oil and gas produced water. Exposed mussels showed accumulation of Ba in the soft tissue several hundred times above background water concentrations and increased concentrations of Sr. Cyclic hydrocarbons were detected in dosed mussels and principle component analysis of gas chromatograph time-of-flight mass spectrometer results could be a novel tool to help identify areas where aquatic organisms are impacted by oil and gas produced water, but larger studies with greater replication are necessary to confirm these results.

## Introduction

Worldwide, an estimated 3 barrels of water are generated for each barrel of oil extracted^1^. Over 3 billion m^3^/year of oil and gas produced water is generated in the United States while pressure to beneficially use this water for agriculture and livestock is growing in water-stressed areas^2–4^. Produced waters often contain elevated radioactivity, total dissolved solids (TDS), dissolved metals, and organics that create unique challenges for disposal or beneficial reuse^5–13^. Accidental spill events, spreading on roads, and permitted releases of treated produced water have led to water and sediment contamination and raised concerns regarding potential toxicity to aquatic organisms^12–29^ even at low concentrations (0.04–3.9%)^22–26^.

A limited number of studies have examined the lethal concentrations (LC_50_) and the developmental, mobility, and transcriptional responses to xenobiotic metabolomics pathways^22–25^ for select aquatic species from exposures to produced water. Acute and chronic effects have been observed in the aquatic invertebrate *Lumbriculus variegatus *(Californian Blackworm) at 10% and 0.1% additions of produced water, respectively. High salinity and low dissolved oxygen were thought to drive the toxicity. Similarly, salinity rather than organics in produced water controlled the mortality (and immobility) of the crustacean, *Daphnia*, introduced to freshwaters mixed with small percentages of raw and synthetic produced waters. Evaluations of zebra fish^12,24,30,31^ and rainbow trout^26,32^ also confirm toxicity and mortality with exposure to low concentrations of produced water. While many of these organisms listed above demonstrate acute and chronic toxicity after exposure to produced waters, the organisms’ mobility in aquatic ecosystems limits their ability to track pollution events or to be indicators of local changes in water quality. In contrast, sessile aquatic organisms, like freshwater mussels, spend their adult lives in one location, thereby allowing them to be potential indicators of water quality in specific geographic locations.

A limited number of studies have examined freshwater mussels exposed to high salinity water. Unionid mussels (*Lampsilis Siliquoidea*) exposed to elevated salinity in the form of high Cl concentrations from KCl and NaCl salts or synthetic inorganic mixtures that represent produced waters resulted in increased mortality and decreased growth^33–35^. In cages placed downstream of an oil and gas discharge, high mortality was observed in riffleshell mussels (*Epioblasma torulosa rangiana*)^36^. Another study demonstrated that freshwater mussels downstream of produced water discharges incorporated strontium into their shell material with ^87^Sr/^86^Sr signatures similar to that measured in oil and gas wastewaters^37^. Importantly, the potential of sentinel species to accumulate produced water contaminants in soft tissue, which could then be used as forensic tools, has not been assessed.

Sampling freshwater mussels, which are sessile, benthic filter-feeders, may be useful to assess water quality and bioaccumulation of environmental pollutants^38–41^. Environmental contamination is proposed as a factor in the recent decline of many freshwater species^42–44^ and the concentrations of trace metals (i.e., Cu, Fe, Mn) in mussel tissue are generally correlated to the concentrations in the water that they inhabit^45–47^. Importantly, studies on freshwater mussels indicate that the metal to calcium ratio in the water and the solubility of the divalent metal hydrogen phosphate mineral controls the accumulation potential of divalent metals in the soft tissue^48–50^. Alternatively, metals could accumulate as secondary precipitated minerals, such as barite in the carbonate shell^51^. Recent research has focused on freshwater mussel species as biomonitors, including monitors of energy extraction activities^34,38,39^ and long term studies have also been conducted such as a study by the National Oceanic and Atmospheric Administration (NOAA) monitoring mussel bioaccumulation of polycyclic aromatic hydrocarbons (PAHs)^41^. The large geographic range of some mussel species makes these organisms candidates for studying the impacts to surface water from energy extraction activities. Importantly, North American freshwater mussel species are undergoing the largest extinction rates compared to any other faunal groups^52–54^.

Here, we exposed adults of a freshwater mussel species, *Elliptio complanata,* to low concentrations (1–4%) of hydraulic fracturing produced water similar to concentrations that were reported downstream of oil and gas wastewater disposal facilities. We assess the potential contaminant accumulation in adults in a limited controlled laboratory study to determine possible forensic tracers. We hypothesized that exposure to produced water can result in the accumulation of constituents in both the soft tissue and the hard shell that can be used to fingerprint the source of contamination events.

## Results and discussion

### Incorporation of organic signatures

**Figure 1:**
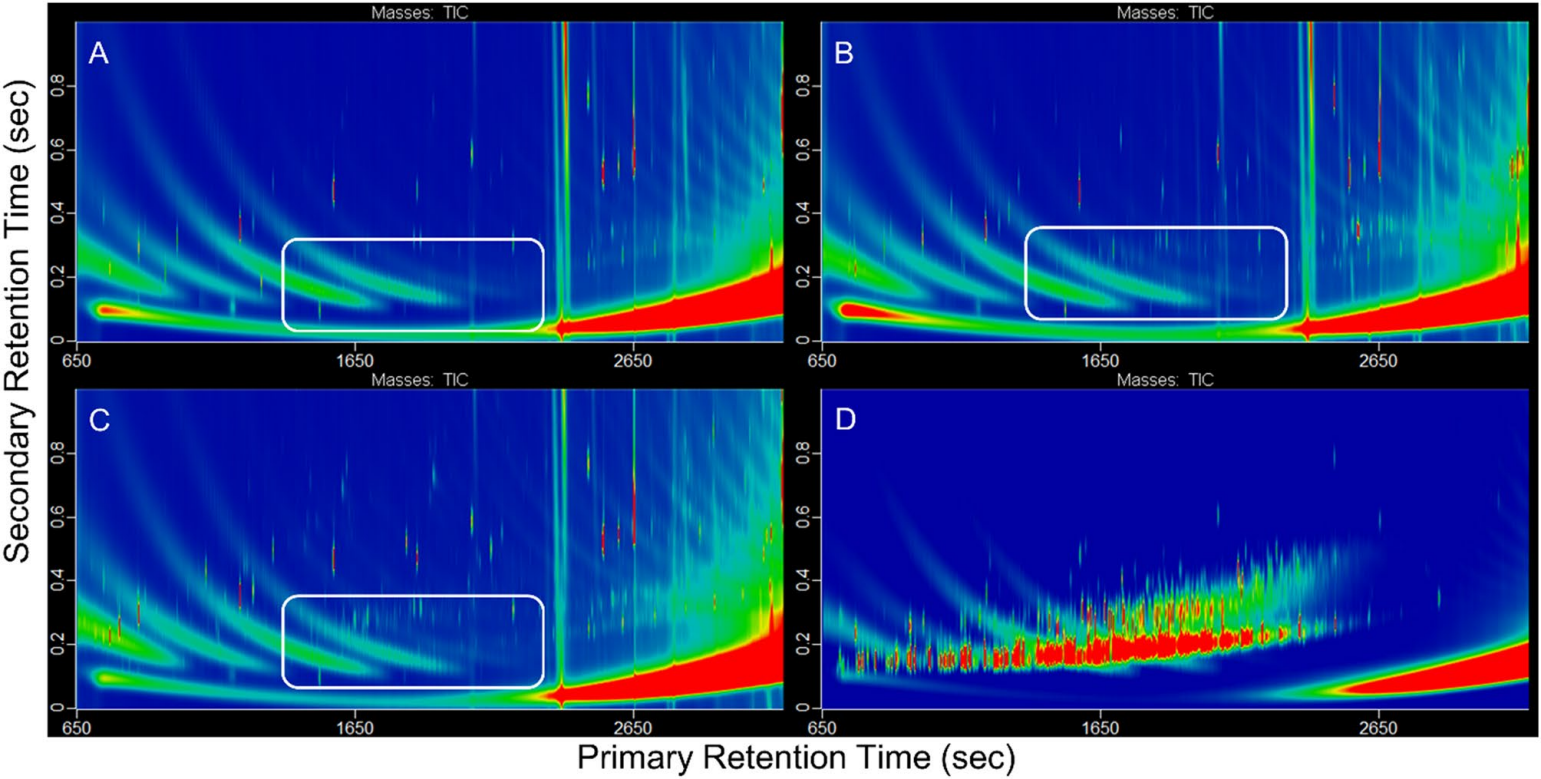
The GC × GC-TOFMS total ion chromatograms for fatty tissue of mussels exposed to produced water from hydraulic fracturing operations (**A**) control group, (**B**) low-dose, (**C**) high-dose (**D**) the produced water used for the dosing. The mussels exposed to produced water (panel **B** and **C**) show an increase in cyclic hydrocarbons as observed in the produced water panel (**D**) as highlighted in the white ovals. The produced water (**D**) is dominated by saturated hydrocarbons, including n-alkanes, branched alkanes, and cyclic saturated hydrocarbons.

Bioaccumulation of organic constituents in the fatty tissues of mussels was assessed by analysis with GC × GC-TOFMS, an analytical technique that maximizes the number of separated chemical compounds in a single analysis. This chromatographic method separated hydrocarbons based on vapor pressure (boiling point) in the x-axis and by pi-pi intermolecular interactions in the y-axis. Since compound separation is performed through interactions with the stationary phase of the columns, the axes are thus labeled retention times to indicate when the separated compounds elute from the columns. Since two mechanisms of separation are employed in GC × GC, compounds that are chemically related (i.e. increasing carbon chain lengths) elute in diagonal patterns in the chromatographic space. Differences in GC × GC-TOFMS chromatographs between treatment groups (control, low and high dose) show that hydrocarbons in oil and gas produced waters accumulated in the mussel tissue. Compared to mussels grown in control tanks (Fig. 1A) mussels exposed to produced water showed a higher abundance and diversity of cyclic hydrocarbons such as cyclopentanes, cyclohexanes, and hydronaphthalenes (white ovals in Fig. 1B,C). These signatures in fatty tissue are very similar to the GC × GC-TOFMS chromatographs of produced water, which is dominated by saturated hydrocarbons, including n-alkanes, branched alkanes, cyclohexanes, cyclopentanes, adamantanes, and hydronaphthalenes (Fig. 1D). While not every compound present in the produced water is also present in the mussel tissue, it is likely that the higher abundance and diversity of cyclic hydrocarbons observed in the fatty tissues is a result of the exposure to produced water.

**Figure 2:**
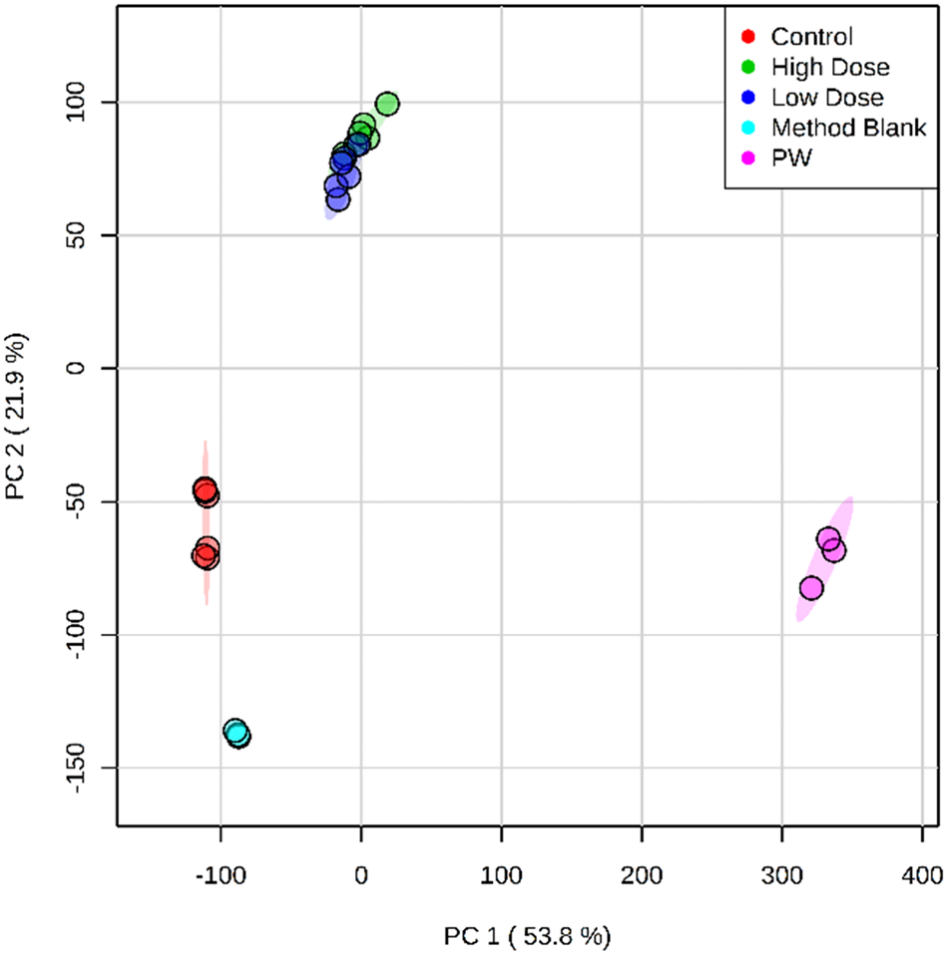
The principle component analysis score plot of aligned GC × GC-TOFMS peak lists from produced water (pink circles) and fatty tissue of mussels exposed to both high (green circles) and low doses (blue circles) of the same produced water from hydraulic fracturing operations. Fatty tissue from mussels that were not exposed are shown in red circles, and method blanks are in light blue circles. Note that the exposed fatty tissue samples score separately than both the unexposed control tissue samples and method blanks. Over 70% of the score difference between the control and the exposed mussels is attributable to cyclic hydrocarbons. Analytes detected by GC × GC-TOFMS were subjected to Kruskal–Wallis ANOVA and p < 0.05 was required for PCA modeling; shading represents the 95% confidence interval.

To determine if the cyclic hydrocarbons found in the fatty tissue of mussels that were exposed to produced water are statistically differentiated from the control group, a principal component analysis (PCA) of the GC × GC-TOFMS data was performed^55^. Both the high and low dose exposure groups plotted away from both method blanks and the control samples as well as produced water (Fig. 2). The PCA features that contributed to the differentiation between dosed and un-dosed groups were attributed to exposure to produced water because of the shift in both PCA 1 and PCA 2 scores. Of these features, 74% were cyclic hydrocarbons, which is a major class of compounds in the produced water. The persistence of the cyclic hydrocarbons in the fatty tissue may be a potential fingerprinting mechanism for identifying shale gas impacts. Aquatic organisms are known to accumulate PAHs, which can result in negative health responses^56^. Studies on a predator of mussels, muskrats, has shown that populations living in areas with higher PAHs in sediment could be negatively impacted^57^.

Analyzing the PCA, it is important to note that exposed mussels did not plot in a region near the produced water, indicating the produced water is chemically distinct from the mussels. However, the mussels exposed to produced water are more chemically similar to the produced water than those that were not exposed to the produced water, as shown by the separation between unexposed and exposed mussel populations in PC1 (Fig. 2). In addition, the exposed mussels show a large difference from the control mussels, as shown in PC2. Combined, the method indicates a measurable difference in exposed populations, as determined by PCA.

*Elliptio complanata* have been used as a biomonitoring species for a diversity of organic compounds. Most commonly, the bioaccumulation of persistent organic pollutants such as polyaromatic hydrocarbons (PAHs) have been studied^58–61^. Previous studies have also assessed the accumulation of other environmental pollutants such as pesticides and polychlorinated biphenyls^62–66^. Sabik et al.^67^ reported on the bioconcentration of alkylphenol polyethoxylates in *E. complanata,* which are common surfactants used in hydraulic fracturing. While we did not observe alkylphenol polyethoxylates in the produced water used in this study, the bioaccumulation of these compounds further supports investigation into the biomonitoring potential of this species from impacts of oil and gas extraction activities.

Laboratory studies have previously determined that unresolved complex mixtures of hydrocarbons, similar to those found in the produced waters used to dose tanks in this study, can be toxic to marine mussels^68,69^. In this work, between 80–100% of the mussels in tanks dosed with produced water died after 56 days, compared to 25% of mussels in the control tank. It is possible that organic compounds in hydraulic fracturing produced water influenced the health and mortality of the mussels. But, due to the chemical diversity of cyclic hydrocarbons in the produced water we cannot attribute the toxicity to any one specific compound present in the produced water. Additionally, it is possible that the observed toxicity may be due to additive or synergistic effects of the mixture of compounds as opposed to individual components^68,69^ or inorganic salt concentrations alone. Indeed, mussel survival is known to be affected by the salts and inorganic compositions of diluted produced water^34^. In freshwaters mixed with small percentages of synthetic produced waters, mussel survival was significantly reduced in waters containing 2,050 and 3,250 mg/L Na and mussel shell length decreased when only 804 mg/L Na was present in the water^34^. Na concentrations in tank waters in this study were much lower, ranging between 398 to 1,824 mg/L in the low and high dose tanks. Yet, our results corroborate previous studies that showed high mortality of freshwater organisms with exposure to relatively dilute (1–5%) concentrations of produced water. While the work herein did not discriminate between mortality caused by salinity or organic compounds (e.g., with salt water or organic controls), future studies could focus on the mechanism of mortality.

### Incorporation of inorganic constituents

The potential for freshwater mussels to accumulate metals over a relatively short exposure period (56 days) was quantified using both the accumulation factor (AF) and metal accumulation relative to calcium. The AF was calculated for each metal based on the final concentrations of metals measured in the soft tissue divided by the concentration in the water—but the authors note these may not represent equilibrium conditions. In addition, metal signatures in the carbonate shell were analyzed to determine if both shell and tissues could be used in forensic applications for relatively short exposure times. The accumulation factor calculated for *Elliptio complanata* varied widely between metals analyzed (Cu, Mn, Mg, Ba, Sr) (Table 1). Accumulation factor values > 1 were calculated for Cu, Mn, Mg, Ba, and Sr which indicates magnification of metal concentrations from water to soft tissue during exposure. The higher concentrations observed in the soft tissue could indicate the use of Cu, Mn, Mg, Ba, and/or Sr as candidates for forensic tracing of produced water exposure.

**Table 1:** Water and soft tissue concentrations for control high and low dose tanks.

Water (mg/L)	Sr	Mn	Mg	Ca	Fe	Ba	Cu	Na
Utica Brine	2,990	BDL	1980	17,200	146	372	BDL	34,700
Control	0.1	BDL	20	49	BDL	0.1	BDL	51
LD Early*	30	BDL	40	220	BDL	3.8	BDL	398
LD Late	72	BDL	72	450	BDL	8.9	BDL	946
HD Early*	75	BDL	69	478	BDL	9.4	BDL	917
HD Late	151	BDL	116	845	BDL	18.1	BDL	1824
**Water (mmol/mol[Ca])**								
Control	0.001	BDL	1	1,000	BDL	0.36	BDL	NM
LD Early*	0.3	BDL	2	1,000	BDL	5.01	BDL	NM
LD Late	0.8	BDL	3	1,000	BDL	5.77	BDL	NM
HD Early*	0.8	BDL	3	1,000	BDL	5.72	BDL	NM
HD Late	1.7	BDL	5	1,000	BDL	6.26	BDL	NM
**Soft tissue (mg/kg)**								
Control	427	18,564	544	38,982	24,088	93	30.1	NM
LD Early*	1822	19,379	622	44,129	29,755	1,258	13.9	NM
LD Late	2,134	18,759	836	46,914	26,388	3,875	34.0	NM
HD Early*	1882	10,504	820	31,560	16,764	1856	28.6	NM
HD Late	2,308	23,524	778	54,491	34,999	5,705	17.1	NM
**Soft tissue (mmol/mol [Ca])**								
Control	5	348	23	1,000	444	0.7	0.49	NM
LD Early*	19	321	23	1,000	484	8.3	0.20	NM
LD Late	21	292	29	1,000	404	24.1	0.46	NM
HD Early*	27	243	43	1,000	382	17.2	0.57	NM
HD Late	19	315	24	1,000	461	30.6	0.20	NM
**Accumulation factor**								
Control	3,866	NC	27	799	NC	1533	NC	NC
LD Early*	61	NC	16	200	NC	333	NC	NC
LD Late	30	NC	12	104	NC	435	NC	NC
HD Early*	12	NC	7	66	NC	102	NC	NC
HD Late	31	NC	11	64	NC	610	NC	NC
**Distribution coefficient**								
Control	4.84	NC	0.03	1.0	NC	1.92	NC	NC
LD Early*	0.30	NC	0.08	1.0	NC	1.66	NC	NC
LD Late	0.28	NC	0.11	1.0	NC	4.18	NC	NC
HD Early*	0.33	NC	0.19	1.0	NC	2.74	NC	NC
HD Late	0.27	NC	0.10	1.0	NC	5.34	NC	NC

*BDL* below detection limit, *NM* not measured, *NC* not calculated, *HD* high dose, *LD* low dose. *Concentration in water estimated from mass balance of Utica brine and Control water- all other concentrations were measured.

#### Strontium, barium and magnesium

**Figure 3:**
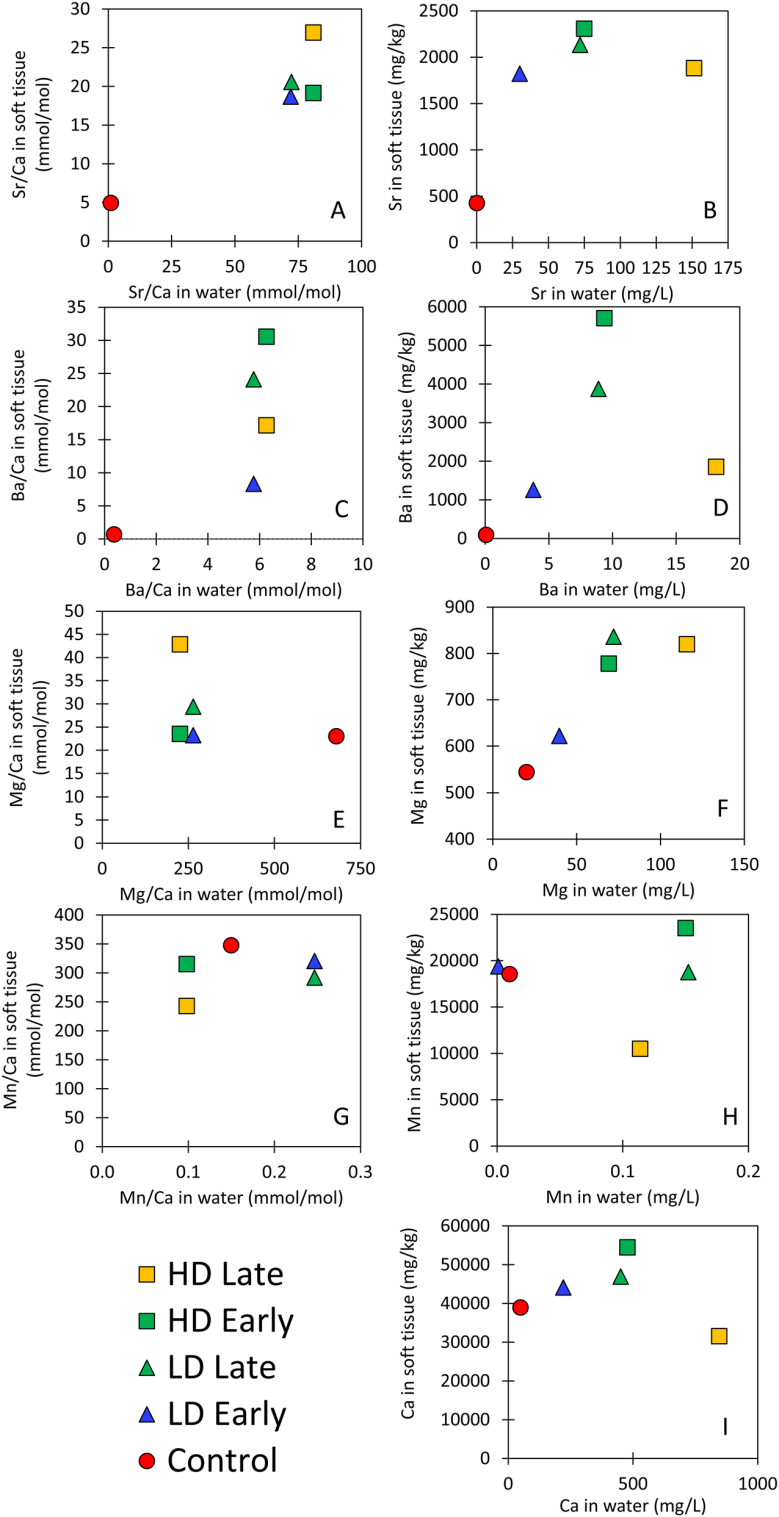
Metal (Sr, Ba, Mg, Mn, Ca) to calcium ratios and metal concentrations (mg/kg) in the soft tissue of freshwater mussels compared to the values in the water for control (red circle) and samples in the high dose (squares) and low dose (triangles) tanks collected both early (~ 4 weeks [green square and blue triangle) and late (~ 8 weeks following a second dose [yellow square and green triangle]). The color represents the dose of produced water with the highest dose (1:20) represented by yellow, the moderate dose 1:40 (green), and the low dose (1:80) by blue. Metal/Ca ratios in the soft tissue of mussels collected from dosed tanks generally increased relative to the control tanks for both Sr, and Ba (**A**, **C**). However, other metal/Ca ratios such as Mg or Mn (**E**, **G**) did not show a clear relationship. For example, Mn/Ca ratios in the soft tissue were highest in the control tank mussels. Mn/Ca_tissue_ displayed a slight negative trend with dose and exposure time. Mg concentrations in the soft tissue appear to be related to concentration in the water without apparent influence from ratios with calcium, but similar to both Sr and Ba, the relationship may not be as strong at the extreme concentrations observed in the highest dose of produced water.

The accumulation factor calculated for Sr was > 1, indicating the mussels incorporated Sr from the water into the soft tissue. There was both a positive correlation between the Sr concentration in the water and in the soft tissue and between Sr/Ca ratios in the water (Sr/Ca_water_) and tissue (Sr/Ca_tissue_) (Fig. 3), indicating magnification of Sr in dosed tanks (AF_Sr_ = 12 to 31). Despite the accumulation of Sr, mussels showed a preference to incorporate Ca in the tissue relative to Sr with distribution coefficient (D_Sr_) values of 0.27 and 0.33. These D_Sr_ values < 1 indicate preferential uptake of Ca into the soft tissue compared to Sr, when both elements are present in solution. Indeed, the highest concentrations of Sr were present in the mussels collected late in high dose tank (151 mg/L), but similar concentrations of Sr in the soft tissue (~ 1,822 to 2,308 mg/kg) and Sr/Ca_tissue_ (19 to 27) were recorded in all mussels exposed at both low and high dose. This likely reflects the similar Sr/Ca_water_ ~ 75 that was recorded in all dosed tanks.

Ba/Ca and Ba concentrations measured in the soft tissue were 10–60 times higher in the tissue of mussels grown in tanks dosed with produced water compared to controls (Fig. 3). The AF calculated for incorporation of Ba into *Elliptio complanata* soft tissue was between 102 and 610 in the dosed tanks and 1533 in the control tank. Similarly, Ba/Ca was higher in the soft tissue than the water, with D_Ba_ values between 1.66 and 5.34. These results differ from previous studies on a marine mussel species, *Mytilus Edulis*, that showed barium accumulates in soft tissue from either barite formation in the tissues or ingestion of barite that formed in the water column and biomagnification factors (BMF) < 1^70^. The higher AFs (102–610) observed here may indicate that our mussels did not reach equilibrium with the concentrations of the water and the apparent magnification in soft tissue is temporary. Regardless, the Ba/Ca (mmol/mol) of both dosed tanks was very similar to the ratio in the produced water (~ 6) and different than the control water (0.36). Both the Ba AF and the D_Ba_ increased between early and late samples in both high and low dose tanks. The higher Ba/Ca ratios measured in the soft tissue could be a potential sensitive tracer of produced water spills or releases.

The mussels grown in tanks with a moderate dose (high dose early) of produced waters had higher Ba and Ba/Ca in the tissue than the tanks with the lowest dose (low dose early), but mussels exposed to the highest dose (high dose late) did not lead to higher Ba/Ca or Ba in the soft tissue. Instead, both the Ba/Ca and the Ba concentrations in the highest exposure group were intermediate between the lowest dose and the moderate dose for either 28 (high dose early) or 56 day exposures (low dose late). This implies that mussels may sequester Ba in environmental systems where produced waters are released, but the responses are not linear. For example, previous work suggested higher Ba/Ca may be a result of the ingestion of Ba-rich particulate matter or soluble Ba during respiration or feeding large volumes of water^70,71^ and is perhaps excreted at higher doses observed in our study. Laboratory or field studies with longer exposure times are necessary to determine the equilibrium accumulation factors.

Mg_tissue_ increased as a function of Mg_water_ and was higher in the mussels exposed for longer periods of time (56 days). However, this led to Mg/Ca_tissue_ ratios negatively correlated with Mg/Ca_water_. The Mg/Ca_water_ was highest in the control tank and lowest in the high dose tank, meaning the Mg/Ca of the dosed water was lower than control water. This is likely due to the very high calcium concentrations found in produced water^72,73^. D_Mg_ values in the dosed tanks were below 1, meaning Mg was not preferentially accumulate relative to Ca. D_Mg_ values were higher in mussels exposed for the full 56 days compared to 28 days. The accumulation factor was less than 1, meaning Mg did not biomagnify in the mussels, although a higher Mg_water_ corresponded to higher Mg_tissue_ and was highest in the mussels exposed for longer time periods. Mg incorporation into the shell is thought to be indicative of stress^74,75^, but Mg incorporation mechanisms in the soft tissue of freshwater mussels have not been described in detail. In either scenario, the lower Mg/Ca ratios in produced water and lack of background studies on Mg incorporation indicate that Mg may not be a sensitive tracer of produced water.

#### Calcium, manganese, iron, and copper

The produced water used in this study contained very high concentrations of calcium (17,200 mg/L). A positive relationship between concentration in the tank water and the concentration in the soft tissue was observed for all samples other than the day 56 sample in the high dose tank (high dose late). This may indicate that the high dose late specimens were no longer filtering. Indeed, the high dose late tissue sample concentrations for Sr, Ba, Mg, Mn and Ca, were consistently lower than most of the dosed tank samples. In addition, a negative relationship was observed between calcium concentration in the tank and the accumulation factor (Fig. 3I).

The concentrations of Mn, Fe, and Cu observed in soft tissue from both control and dosed tanks is consistent with a mechanistic control from Me^2+^-phosphate solubility for the accumulation of divalent metals in soft tissue of freshwater mussels as reported for other species^49^. Mn_water_ was below detection in both the control sample and the produced water, therefore, Mn may offer limited value as a forensic tracer of exposure. Despite the low concentrations of Mn measured in the water, the elevated Mn/Ca measured in the tissue appeared to demonstrate that Mn would likely produce both a large accumulation factor and a D_Mn_ > 1 that may be related to the extracellular formation of the relatively insoluble Mn-hydrogen phosphate^49,50^. Likewise, Fe concentrations in the water samples were below detection limit, but higher concentrations (13,764–34,999 mg/kg) were observed in the soft tissue indicating a positive and high accumulation factor. Cu appeared to behave in a similar way to Fe and Mn, with rather consistent concentrations observed in the soft tissue regardless of dose. However, Cu was consistently not detected in the water samples and was detected at much lower concentrations in the soft tissue (13.9–34.0 mg/kg) relative to either Mn or Fe.

### Signatures in carbonate shells

Shells of mussels can also be used as an indicator of water quality. Mussels grow a shell of calcium carbonate in sequential layers through biomineralization in the extrapallial fluid^76^. While this fluid is isolated from the surrounding water, the mollusk derives the material used to build its shell from its environment, entering the hemolymph through respiration, ingestion, and direct uptake by the outer epithelium^77^. Transport of elements through the mussel are mediated by transport enzymes, which exert selective control on what elements enter the extrapallial fluid^78^ where organic macromolecules mediate the precipitation of calcium carbonate (CaCO_3_), building an aragonite or calcite structure bound by an organic framework^79,80^. As a consequence, shell precipitation can reflect environmental conditions but does not follow the expected thermodynamic partitioning behavior of inorganic minerals^81^.

Ablations of the hard calcium carbonate shell material did not show any significant indications of changes in concentrations in the distal 2 mm of shell, indicating that shell growth likely did not occur during the study period (56 days). This is not surprising given the high mortality. Unfortunately, this limits the utility of the shell material in forensic studies where high mortality was observed, but the utility in low-mortality areas is still promising. In high mortality conditions, only the soft tissue collected at the time of mortality is likely useful to fingerprint the contaminant source. Notably, the laser ablation method would likely not detect small barite minerals excreted from the extrapallial fluid onto the inner shell layer, as previously observed^44^.

### Comparison to previous studies

The elevated TDS and Cl concentrations are known to cause high mortality among juvenile freshwater mussels^36^. The chloride concentrations in this study (low dose = 1,240 and high dose late = 4,470 mg/L) fall within the ranges (1,026–5,190 mg/L) used by previous studies to evaluate mortality associated with salinity^34^. Chloride concentrations were above the short-term aquatic toxicity for chloride (640 mg/L) as applied broadly to aquatic organisms by Canadian Water Quality Guidelines^82^. Both the short duration of the experiment and the use of a limited number of adult specimens for each exposure limits the conclusions that can be drawn regarding mortality rates (e.g., LC50 values). The influence of salt content or duration of exposure on mussel mortality could not be determined due to the similar mussel response in both the low and high dose treatments, but previous studies demonstrate the importance of both chloride and water hardness when assessing mortality^33–35,83,84^. Because our study utilized a relatively small number of individuals, a different genera, and adult mussels compared to previous studies, mortality rates were not thoroughly evaluated. Regardless, mortality rates here were similar to field studies^36^ (See Supplemental Material).

The potential for cyclic hydrocarbons to be used as forensic tracers through the food web is an area of potential future study. Studies on a major predator to mussels, muskrats, who lived were PAHs accumulated in sediments showed an association with negative health outcomes[^85^]. Our results are consistent with previous studies that demonstrated *Elliptio complanata* had the potential to bioaccumulate PAHs^58–61^ and now a PCA approach could potentially fingerprint the accumulation other organisms.

## Conclusions

Exposing freshwater mussels to oil and gas produced water showed the potential of produced water constituents such as Ba and Sr to accumulate at concentrations in soft tissue that could be used as forensic tools to identify releases to surface water. Overall, prolonged exposure to produced water resulted in 1) an increase in barium and cyclic hydrocarbons concentrations of soft tissue, and 2) incorporation of potentially unique tracers of cyclic hydrocarbons, Ba, and Sr that were distinguishable from signatures observed in control samples. As a forensic tool, petroleum hydrocarbons, Ba and Sr, are of particular interest because they are in high concentration in oil and gas produced water and often contain unique isotopic or element ratios. This work demonstrates that cyclic hydrocarbons and Ba can accumulate at concentrations 100–300 times that of the water. This work also suggests that mussels may be a significant end point when studying the fate of Ba downstream of permitted oil and gas releases and *may* indicate that studies of oil and gas produced water discharges should evaluate species that readily consume mussels such as muskrats, otters, raccoons, crayfish, birds, and fish for potential impacts^56,57^.

The study presented herein was designed to determine if *Elliptio complanata* mussels could be used as biomonitoring species, recording changes in freshwater from releases of oil and gas produced water at concentrations similar to those observed downstream of permitted facilities. Additional research is necessary to more accurately quantify the relationship between water chemistry and soft tissue, establish equilibrium constants (e.g. BMFs and chemical elimination rate constants), and to demonstrate the capability of mussels to record chemical signals of produced water in a natural system. Specifically, quantifying individual bioaccumulated organic compounds will aid in determining the cause of toxicity when exposed to produced water, which would be beneficial in assessing the broader impacts that contamination events can have on mussels. With further study, it is possible that mussels may also be used as sensitive indicators of contamination associated with releases of oil and produced waters.

## Materials and methods

### Tank parameters

Both medium and large (5–7 cm length) adult *Elliptio complanata* were purchased from Carolina Biological Supply Company (Burlington, NC). The mussels were kept in 50 L aquaria filled with University Park, PA tap water that was preconditioned (i.e., aged to allow the reduction in chlorine) for 14 days prior to use with the mussels. Eight to nine mussels (n = 8 for low dose and control, or n = 9 for high dose) were kept in each tank and water was maintained at 21–22 °C with pH 7.2–8.2 throughout the study. Mussels were fed daily with 5 mL of algae food source obtained from Carolina Biological Supply Company, Burlington, NC. Ammonia and pH were measured frequently during the study. Mussels grew for a maximum of 60 days prior to harvesting. Tanks were exposed to laboratory lighting for roughly 12 h periods followed by 12 h of darkness. Bioaccumulation and toxicity were assessed for three treatment levels: control, low-dose, and high-dose, each with 8–9 individuals per dose. Produced water for the dosing was obtained from a hydraulically-fractured Utica Shale well located in southeastern Ohio. Concentrations of select constituents are presented in Table S1. The control tank contained only conditioned tap water while the low-dose contained a 1:80 dilution of produced water, and the high-dose contained a 1:40 dilution of produced water. The tanks were re-dosed with produced water after 4 weeks producing final ratios of 1:40 (low) and 1:20 (high). The resulting concentrations mimic those observed 30 to 500 m downstream of centralized waste treatment facilities where changes in water and sediment quality from disposal were observed previously^86^. The dilutions were determined based on the total organic content (TOC) values reported in streams impacted by unconventional gas development and the measured TOC in the produced water^5,6^. Importantly our study mimicked the concentrations in a static manner, but not the daily cycle of high and low concentrations observed downstream of facilities or lower concentrations that might be observed longer distances downstream where larger dilution factors decrease the forensic signals. Mussel mortality was assessed daily with visual inspection. Ammonia was monitored daily and the concentrations controlled with aeration. When mussels died, as assessed by open shells, they were removed from the tanks and stored at − 20 °C until processing.

### Mussel tissue preparation

Whole mussel tissues were dried in a Speedvac until constant weight was achieved. There was no separation of body parts. Thereafter, 0.5 g of dried tissue was transferred to a 10 mL Teflon vial with 5 mL of 2 N nitric acid and 20 µL of hydrogen peroxide. Vials were heated at 60 °C until dry (approximately 6–8 h). This process was repeated 2 more times to oxidize most of the organic matter. Thereafter, the sample was refluxed in 5 mL of 2 N nitric acid for 3 h at 80 °C. The refluxed samples were then diluted to 40 mL with 2 N nitric acid and 4 mL of 12 M HCl. The diluted samples were further digested in a CEM Mars 6 microwave digester at 200 °C and 26.6 bar (400 psi) for 40 min. Microwave digested samples were diluted 100 times in 2% nitric acid before analysis on a Thermo Scientific iCAP 6,520 inductively coupled plasma optical emission spectrometer (ICP-OES, Na, Ca, Mg, Sr, K) or Thermo X-Series 2 mass spectrometer (ICP-MS, Cr, Mn, Fe, Co, Ni, Cu, Ba, Pb). All dilution factors were accounted for on a mass basis and elemental concentrations were normalized by mass of tissue digested.

Mussel tissue was prepared for organic analysis following the Extraction of Biological Tissue or Trace Organic Analysis procedure outlined in the NOAA Mussel Watch program^41^. For analysis, mussels were pooled for analysis based on time of death, and separated into 2 groups: early, and late mortality. Within each group, the soft tissue of two individuals was combined to generate enough material to analyze and one value was reported. For example, in the high dose tank, the tissue of mussels 7 and 8 were combined prior to analysis. Approximately 3 g of homogenized fatty tissue was mixed with 10–15 g of sodium sulfate and tissuemized for 3 min with three aliquots of 25 mL of dichloromethane. The samples were concentrated to 1 mL using a Kuderna-Danish apparatus and a sample cleanup was performed with 50 mg of primary-secondary amine media to remove the fatty acids native to mussel tissue. The samples were analyzed using GC × GC-TOFMS following the method described above with a 1:2 injector split.

### Organic and inorganic measurements

The produced water sample was prepared for organic analysis by liquid–liquid extraction using a modified version of USEPA Method 3510C^87^. The produced water (500 mL) was extracted 3 times with 25 mL of dichloromethane and concentrated to 1 mL using a Kuderna-Danish apparatus. Produced water and soft tissue were analyzed using GC × GC-TOFMS (LECO Pegasus 4D, St. Joseph, MI), which was operated with a 250 °C injector temperature and a 2 mL/min helium flow rate. The first-dimension column was a Restek Rtx-DHA100 (100 m × 0.25 mm × 0.5 μm) (Bellefonte, PA) and the second-dimension column was a Restek Rxi-17SilMS (1.0 m × 0.25 mm × 0.25 μm). The first-dimension oven was programmed to maintain 50 °C for 0.2 min after a 1μL 1:100 split injection and increase at 5 °C/min to 315 °C. The modulator temperature was offset at 15 °C positive, and operated with a 2 s modulation period. The second-dimension oven was offset by 5 °C. The mass spectrometer operated with a 700 s acquisition delay, 250 °C ion source temperature, and − 70 eV ionization energy. The collected mass range was 30–550 amu at 200 Hz.

The produced water sample was prepared for inorganic analysis by acid digestion in accordance to USEPA method 3005A^88^. Major and minor trace element analyses were performed on a Thermo Scientific iCAP 6,520 inductively coupled plasma optical emission spectrometer (ICP-OES, Na, Ca, Mg, Sr, K) and a Thermo X-Series 2 mass spectrometer (ICP-MS, Li, B, Ba, Fe, Mn, Pb, Cd, Cu, As, U). Quality assurance calibrations for all instrument were verified by comparing measured and known elemental concentrations in check standards (USGS M-220, USGS T-227, and SRM1640a).

### Laser ablation of carbonate shell

After the mollusks were harvested and the soft tissue was removed, the shells were prepared for laser ablation. Valves from each shell were cast in a hard, clear epoxy, then sectioned using a low speed mineral saw with a MK diamond MK-303 0.76 mm abrasive cutting wheel. Each puck was then polished on a mineral polishing wheel, cleaned using EMD Millipore Triton X-100 non-ionic surfactant, and washed with ultrapure 18.2 MΩ.cm water.

Polished shell samples were then analyzed for trace and major element chemistry using an ESI NWR193 laser coupled to a Thermo Xseries II ICP-MS located at the Penn State University Energy and Environmental Sustainability Laboratories (EESL). The laser was set to 30% power, a travel speed of 40 um/s, a pulse repetition rate of 40 Hz, and a spot size of 100 μm. A 10 s delay and a 15 s drawdown time were added at the start and end of each ablation. USGS MACS-3, a pressed powder carbonate, and NIST SRM 612, a trace metal in glass standard were ablated at the start and end of the set of ablations to correct for instrument drift and to calibrate the instrument. Metal concentrations are normalized to Ca, which acts as an internal standard to account for differences in ablation behavior between samples and standards.

### Statistics

The potential accumulation of metals in the mussel soft tissue was calculated by dividing the concentration measured in the dry tissue (mg/kg) by the concentration measured in the water (mg/L) to establish accumulation factors. These factors are analogous to biomagnification factors (BMF) or concentration factors, but importantly are not true BMFs because we did not assess if the concentrations in the soft tissue reached equilibrium. Calcium-normalized molar distribution (D_Me_), in both soft tissue and hard shell was calculated by dividing the Sr/Ca_tissue_ by the Sr/Ca_water_ to determine preferential incorporation of metals relative to calcium.

Statistical analysis was performed using MetaboAnalyst 4.0^89^. The GC × GC-TOFMS data were first subjected to basic data processing in the ChromaTOF software followed by peak alignment and peak wise comparison in the Statistical Compare feature of ChromaTOF. The alignment results were exported to Excel and manual quality control was applied to remove peaks corresponding to column bleed. The data were then subjected to normalization based on an internal standard, mean centering and log transformation in accordance to procedures outlined by Weggler et al.^55^ A non-parametric ANOVA (Kruskal–Wallis) was performed at p < 0.05 to determine the analytes that contributed significantly. A principal component analysis (PCA) was performed on the significant analytes as a non-targeted data analysis tool to determine differences between treatment groups and the features which contribute to the differentiation.

The concentrations of major elements measured in both the low and high dose tanks represent roughly 1:40 and 1:20 mixtures of produced water to control tank water and are consistent with concentrations calculated via mass balance. Concentrations in soft tissue are reported on a dry weight basis (mg/kg). In most cases, individual samples of soft tissue from each tank were combined based on time of harvest to obtain enough material for analysis. Early samples were collected after 28–35 days and late samples after 56 days growing in the tanks.

## Supplementary information

Supplementary file1
