# Supplementary material for: Forensic tracers of exposure to produced water in freshwater mussels: a preliminary assessment of Ba, Sr, and cyclic hydrocarbons
Source: Sci Rep. 2020 Sep 22;10:15416. doi: 10.1038/s41598-020-72014-6 (PMC7508860; doi:10.1038/s41598-020-72014-6)
Supplement: Supplementary file 1 — Supplementary file1 [file 41598_2020_72014_MOESM1_ESM.docx]

**Supplemental Material for:**

**Forensic Tracers of Exposure to Produced Water in Freshwater Mussels: A Preliminary Assessment of Ba, Sr, and Cyclic Hydrocarbons**

Paulina K. Piotrowski,^1^ Travis L. Tasker,^2,3^ Thomas J. Geeza,^2,4^ Bonnie McDevitt ^2^, David P. Gillikin^5^, Nathaniel R. Warner,^2^* Frank L. Dorman ^6^*

1) Department of Chemistry, The Pennsylvania State University, University Park, Pennsylvania

2) Civil and Environmental Engineering, The Pennsylvania State University, University Park, Pennsylvania

3) Environmental Engineering, Saint Francis University, Loretto, Pennsylvania

4) EES-14, Los Alamos National Laboratory, Los Alamos, NM 87544

5) Department of Geology, Union College, 807 Union St, Schenectady, NY 12308

6) Department of Biochemistry, Microbiology and Molecular Biology, The Pennsylvania State University, University Park, Pennsylvania

*Corresponding authors.

Nathaniel R Warner, Department of Civil and Environmental Engineering, The Pennsylvania State University, 212 Sackett Building, University Park, PA, 16802,, Phone 814-865-9423,, Email [nrw6@psu.edu](mailto:nrw6@psu.edu)

Frank L. Dorman, Department of Biochemistry, Microbiology and Molecular Biology, The Pennsylvania State University, 107 Althouse Lab, University Park, PA, 16802,, Phone: (814) 863-6805,, Email: [fld3@psu.edu](mailto:fld3@psu.edu)

KEYWORDS: bivalve, mollusk, brine, barium, strontium, cyclic hydrocarbons

2 figures, 1 table

Table S1. Select chemical concentrations of Utica Produced water dosed into tanks.

| Analyte | Concentration (mg/L) |
| --- | --- |
| TDS | 159,000 |
| Cl | 99,400 |
| Br | 1,140 |
| SO4 | <100 |
| Na | 34,700 |
| K | 681 |
| Mg | 1,980 |
| Ca | 17,200 |
| Sr | 2,990 |
| Ba | 372 |
| Fe | 146 |
| Cu | <0.1 |
| As | <0.1 |
| Cd | <0.01 |
| Pb | <0.1 |

Table S2. Calculated TDS values and Cl for each tank.

| Tank | Cl (mg/L) | TDS (mg/L) |
| --- | --- | --- |
| Low Dose Early | 1240 | 2160 |
| Low Dose Late | 2440 | 4070 |
| High Dose Early | 2440 | 4070 |
| High Dose Late | 4740 | 7760 |
| Utica Produced Water | 99,400 | 159,000 |

**Supplemental Results and Discussion**

**Mortality**

Elevated TDS is known to cause high mortality among juvenile freshwater mussels at concentrations similar to those in this study ^1^ , however, our study utilized a relatively small number of individuals, a different genera, and adult mussels compared to previous studies therefore mortality rates were not thoroughly evaluated. Prior to re-dosing on day 28, all mussel populations appeared to be stable and were observed feeding regularly and moving within the tanks. After the re-dosing, large population crashes were observed. Mortality was less than 25% in all tanks prior to redosing, at which point the dosed tank mortality increased to 80-100% by the end of 56 days. Water quality parameters monitored through the study showed pH remained relatively constant at 7.5 for all tanks, which is typical for wild freshwater mussel habitats^2–4^. Ammonia was lowest in the control tank (0-0.25 mg/L) and variable and highest in the high dose tank (1-8 mg/L). The low dose tank was also variable ammonia from 0-4 mg/L). In both dosed tanks ammonia reached maximum values approximately a week after each dosing, likely indicating the stress of produced water dosing leading to toxicity (Supplementary Material Figure S1). The high mortality after the re-dose may be a result of the high chloride concentrations in the tanks dosed with the produced water or potentially the higher ammonia (or a combination of both) (Supplementary Material Figure S2). Chloride concentrations were above the short-term aquatic toxicity for chloride (640 mg/L) as applied broadly to aquatic organisms by Canadian Water Quality Guidelines^5^. The chloride concentrations in this study (low dose =1,240 and high dose late = 4,470 mg/L) fall within the ranges (1,026-5,190 mg/L) used by previous studies to evaluate mortality associated with salinity^6^. Both the short duration of the experiment and the use of a limited number of adult specimens for each exposure limits the conclusions that can be drawn regarding mortality rates. The influence of salt content or duration of exposure on mussel mortality could not be determined due to the similar mussel response in both the low and high dose treatments, but previous studies demonstrate the importance of both chloride and water hardness when assessing mortality^6–10^. The short duration exposures (<28 days) have been demonstrated to be insufficient to evaluate long-term toxicity. Indeed, both the short duration and the use of adult mussels in our study likely lead to lower mortality than would be observed in long term exposure experiments with juvenile and larval mussels such that our mortality results were not quantified. Additionally, we did not assess a positive control of salinity concentrations so the apparent cause of mortality was not determined. In previous studies of indicator species exposed to oil and gas produced water high rates of mortality were associated with elevated chloride positive controls^6,8^.

**References**

1. Patnode, K. A., Hittle, E., Anderson, R. M., Zimmerman, L. & Fulton, J. W. Effects of high salinity wastewater discharges on unionid mussels in the allegheny river, Pennsylvania. *J. Fish Wildl. Manag.* **6**, 55–70 (2015).

2. Geeza, T. J. *et al.* Controls on magnesium, manganese, strontium, and barium concentrations recorded in freshwater mussel shells from Ohio. *Chem. Geol.* **526**, 142–152 (2019).

3. Carroll, M. & Romanek, C. S. Shell layer variation in trace element concentration for the freshwater bivalve Elliptio complanata. *Geo-Marine Lett.* **28**, 369–381 (2008).

4. Kelemen, Z., Gillikin, D. P. & Bouillon, S. Relationship between river water chemistry and shell chemistry of two tropical African freshwater bivalve species. *Chem. Geol.* **526**, 130–141 (2019).

5. Canadian Council of Ministers of the Environment. *Canadian water quality guidelines for the protection of aquatic life. Water Quality Index*. (2001).

6. Wang, N., Kunz, J. L., Cleveland, D., Steevens, J. A. & Cozzarelli, I. M. Biological Effects of Elevated Major Ions in Surface Water Contaminated by a Produced Water from Oil Production. *Arch. Environ. Contam. Toxicol.* **76**, 670–677 (2019).

7. Wang, N. *et al.* Acute toxicity of sodium chloride and potassium chloride to a unionid mussel (Lampsilis siliquoidea) in water exposures. *Environ. Toxicol. Chem.* **37**, 3041–3049 (2018).

8. Wang, N. *et al.* Evaluation of chronic toxicity of sodium chloride or potassium chloride to a unionid mussel (Lampsilis siliquoidea) in water exposures using standard and refined toxicity testing methods. *Environ. Toxicol. Chem.* **37**, 3050–3062 (2018).

9. Todd, A. K. & Kaltenecker, M. G. Warm season chloride concentrations in stream habitats of freshwater mussel species at risk. *Environ. Pollut.* **171**, 199–206 (2012).

10. O’Neil, D. D. & Gillikin, D. P. Do freshwater mussel shells record road-salt pollution? *Sci. Rep.* **4**, 1–6 (2014).

Figure S1. Ammonia concentration in tanks over the course of the study period.


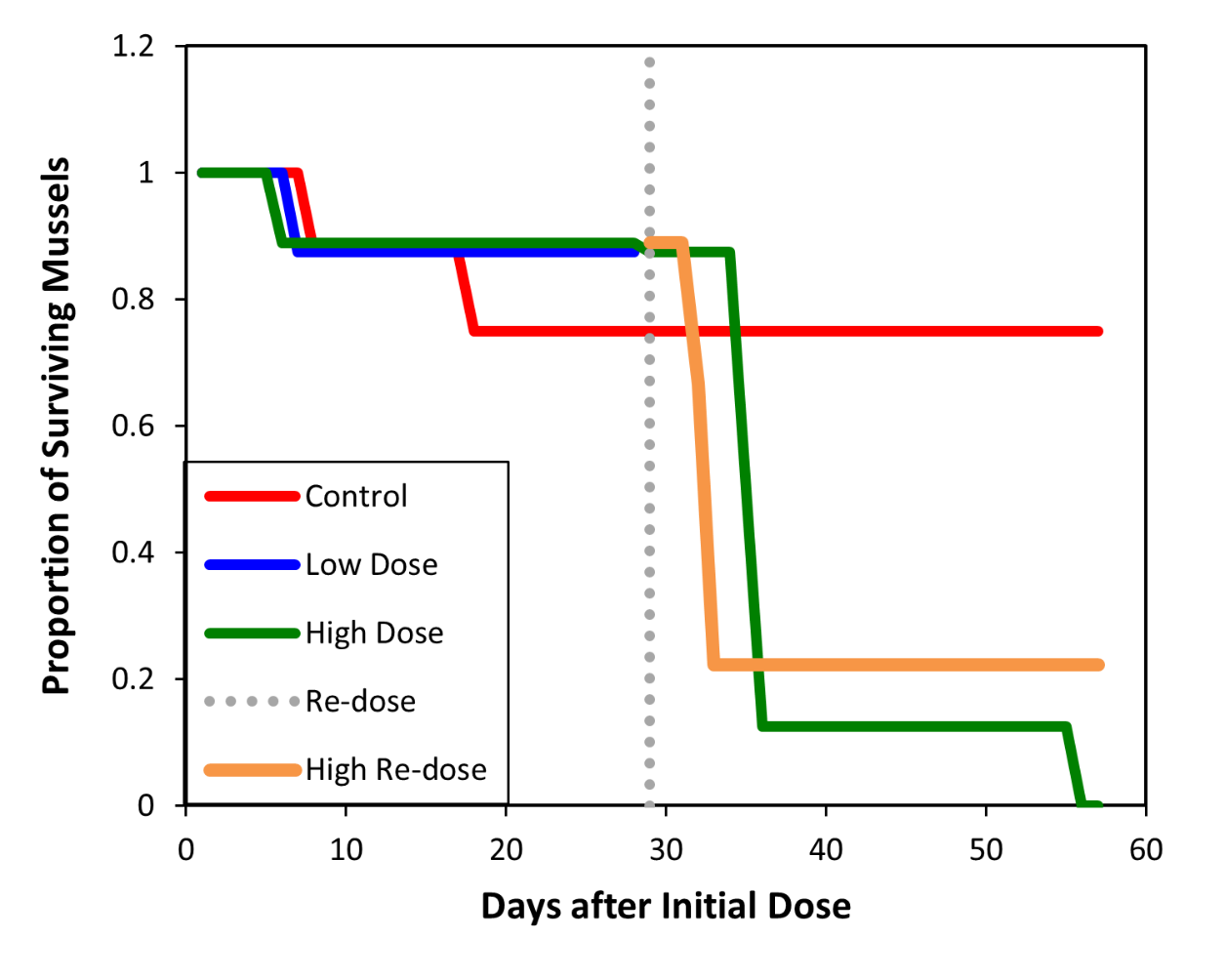


Figure S2. Mortality of freshwater mussels in the three tanks over the course of the study period.
